# The Impact of Generative AI Use on Graduate Students’ Research Competence: The Mediating Role of Critical Thinking and the Moderating Role of Research Self-Efficacy

**DOI:** 10.3390/bs16020304

**Published:** 2026-02-21

**Authors:** Haidong Zhu, Shen Yang

**Affiliations:** Normal College, Shihezi University, Shihezi 832003, China; zhd2005@shzu.edu.cn

**Keywords:** graduate students, generative AI, critical thinking, research self-efficacy, research competence

## Abstract

With the development of the digital intelligence era, generative AI is being widely used in scientific research, and its impact on graduate students’ research competence has attracted much attention from the academic community. Based on cognitive distribution theory and self-efficacy theory, this study classifies AI applications into three levels from basic to advanced—technical support AI use, text development AI use, and transformation AI use—explores their effects on graduate students’ research competence, and examines the mediating effect of critical thinking and the moderating effect of research self-efficacy. The results of the empirical analysis show that all three types of AI use behaviors are significantly correlated with research competence, with the strongest correlation for text development type and the weakest for technical support type. In the relationship between the three types of AI use behaviors and research competence, critical thinking plays a significant positive mediating role, and research self-efficacy plays a significant moderating role. Universities and tutors should guide students to focus on higher-order AI use behaviors in the text development and transformation categories, promoting the use of critical thinking to avoid technology misuse and improving research self-efficacy to help students accumulate confidence and support their research.

## 1. Introduction

As an important force for scientific research in universities, it is crucial to strengthen the development of graduate students’ research competence. With the rapid development of digital technology, artificial intelligence is introducing changes to the research paradigm of scientific research development. Generative AI tools, with their powerful capabilities, such as text generation ability, literature retrieval ability, and code editing ability, enable researchers to greatly improve the efficiency of scientific research, and they play a significant role in data analysis, chart production, literature search and thesis writing (F. Luo & Ma, 2023). Graduate students can use generative AI tools to support their research endeavors. The correct use of AI research tools helps graduate students to optimize their text expression and sentence structure and stimulate creative thinking, thus improving their research efficiency (Jin et al., 2025b). Despite these positive views, some studies have shown that the use of generative AI to obtain information quickly by asking questions lacks deep cognitive processing, critical thinking and other cognitive activities, which may lead college students to develop “technology dependence” and cognitive inertia, and ultimately it is negatively associated with scientific research ability (M. Liu et al., 2025). Therefore, current scholars hold different views on the relationship between generative AI and research competence.

Most scholars in existing research on GenAI usage focus on the following approaches. On the one hand, there are studies on the motivation and willingness to use AI before it is used. Some researchers have categorized AI use into various aspects such as intrinsic motivation, extrinsic motivation, and no motivation to explore the differential relationship between their commitment to research capacity and learning (Wan et al., 2025; F. Li et al., 2025). Other studies have analyzed the factors that influence the adoption of AI technology by users (e.g., teachers, students, or administrators) based on technology acceptance models to explore their willingness to use AI (Saha et al., 2025; Saqr et al., 2024). On the other hand, Pacheco-Mendoza et al. (2023) measured AI use based on the number of days, hours, and the number of tools that students used, focusing on the intensity of the behavior. Khotimah and Rusijono (2024) compared AI feedback tools embedded in students’ daily learning through an experimental pre- and post-test to explore how meta-learning impacts metacognition and creativity through AI use. In summary, existing studies have explored the relationship between AI and students’ ability development mainly in terms of students’ motivation to use AI and intensity of use.

The AI use in this study focuses on the latter, i.e., the systematic behaviors of students when employing generative AI to aid in research. However, the classification framework of generative AI usage behaviors in existing studies is still insufficient. First, the categorization criteria are relatively homogeneous. Previous studies tend to focus only on the cognitive dimension of users or are limited only to the technical implementation and functionality dimension. For example, Joerling (2025) builds a framework from the technical–functional dimension, distinguishes GenAI as a “research method” and “research object”, and explores how GenAI can contribute to marketing research, but the purely technical perspective strips away the intention and cognitive depth behind the behavior. Chan and Lee (2025) integrate cognitive, affective, and metacognitive dimensions into a framework to explore how AI can improve efficiency and reduce cognitive load from a cognitive perspective. However, the framework generalizes usage behavior to the cognitive and affective dimensions, ignoring the other dimensions.

This one-dimensional perspective leads to an incomplete explanatory power of the framework to portray the true dynamics of human–computer collaboration in its entirety. Those who fall under the cognitive label may include both researchers who use prompts to stimulate deeper thinking and students who use AI with purely instrumental attributes of data processing and grammar revision only. The two are in fact fundamentally different in terms of ability development and learning outcomes. Second, most frameworks in existing studies fail to clearly construct an AI-using behavior from passive acceptance to active creation. They tend to synthesize different dimensional categories at the same logical level (Chaieb et al., 2026), thus failing to clearly articulate how these differentiated usage patterns affect the core competencies of the researchers respectively.

As a result, this study categorizes generative AI usage into three types of usage behaviors: technical support AI use, text development AI use, and transformation AI use. Specifically, the relationship between AI use and research competence appears to vary depending on context. According to distributed cognitive distribution theory, cognitive tasks are distributed among internal and external representations. Learning is a process of social interaction and collaboration. Individuals solve problems and construct knowledge systems by communicating and cooperating with others while utilizing external tools (Zhou & Fu, 2002). First, externally, generative AI, as a powerful external cognitive tool, can help learners break through individual limitations to quickly and systematically retrieve, analyze, and summarize large amounts of data (Allen & Mizumoto, 2024). In academic research, tools such as ChatGPT and Elicit are widely used to reduce the burden of information processing and automate repetitive tasks (Khan & Suhluli, 2025). This enables researchers to access and filter information quickly, thereby increasing research efficiency. Second, internally, generative AI is able to generate new content, including text, diagrams, etc., based on human prompts (L. Chen et al., 2025). It helps researchers to bring novel design paradigms (G. Chen et al., 2025) and inspire by facilitating internal representational task processing, such as concept design and idea generation.

According to the theory of self-efficacy, the role of research self-efficacy is mainly reflected in the ability to regulate the individual’s cognition, emotion, choice and effort, which has an important impact on the final behavioral results, and different levels of research self-efficacy can have different relationships with research ability (Greeley et al., 1989).

Based on the theory of cognitive distribution and self-efficacy, this study focuses on three forms of generative AI use: technical support AI use, text development AI use, and transformation AI use. Through delving into the relationship between generative AI applications and research competence, it tests the mediating role of critical thinking and the regulating role of research self-efficacy. It aims to guide technology developers and researchers to jointly explore AI tools that can promote researchers’ deep thinking rather than substitute thinking, and finally it aims to achieve synergy and improve researchers’ research competence.

## 2. Literature Review and Hypothesis Development

### 2.1. Generative AI Use and Research Competence

“AI for Research” (AI4R), as a fifth paradigm of scientific research, emphasizes that AI substantially improves scientific research capabilities through knowledge automation, human–computer intelligence fusion, and the solving of combinatorial explosion problems (G. Li, 2024). Existing studies have shown that in literature search and writing, AI tools improve the efficiency of literature review, making it three to eight times faster than traditional methods (M. Liu et al., 2024). Regarding data visualization, some studies have shown that researchers’ data visualization ability is significantly improved after using AI-assisted systems (Wu et al., 2024; Jadán-Guerrero et al., 2024). However, the relationship between AI and research capacity is complex and dual in nature. Intrinsically motivated AI use is associated with scientific creativity, whereas extrinsically motivated use requires deep cognitive processing to avoid inhibiting creativity (Wan et al., 2025). If the researcher adopts the AI results directly using the default acceptance model, it will contribute to a weakening of innovative thinking. Highly independent researchers can turn AI into “research assistants” and improve their professional well-being by relieving work stress, while conversely increasing anxiety (J. Zhang & Zhou, 2025).

In this study, “generative AI use” refers to the systematic behavior of students in applying generative AI to assist in scientific research. Referring to the dimensions of the existing literature, this study categorizes generative AI use into three tiers: technical support, text development, and transformation (Jin et al., 2025a). Technical support AI use refers to its use for basic instrumental aids. This includes instrumental behaviors such as code writing (Y. Liu et al., 2024), charting (Palaniappan et al., 2025), formatting (Zohery, 2023) and data processing (Allen & Mizumoto, 2024; Hassani & Silva, 2023). The aim is to improve the efficiency of scientific research and it does not involve the creation of theories, ideas, or other content. Text development AI use refers to its use for content generation and expansion. This includes acts such as logical restructuring (H. Jung & Park, 2023), frame generation (Gahar et al., 2025), textual expression optimization, and stylistic transformation (De Wilde, 2024; Kim et al., 2025). On the other hand, transformation AI use goes beyond AI tool attributes. It promotes researchers’ thinking and cognitive reconfiguration through deep interaction with AI, such as disciplinary integration through AI (K. D. Wang et al., 2024), mind expansion (Lingard, 2023; Chia & Frattarola, 2025), and facilitation of research reflection (Chan & Lee, 2025). Examples of each AI use type can be seen in Table 1.

Differences in the relationship between generative AI and research competence may stem from the different depths of human–computer cognitive distribution patterns (An, 2025). From the cognitive distribution theory, generative AI can take on different tasks in the scientific research process and expand the cognitive scope of the researcher. If it is viewed only as a tool (e.g., technical support AI use), the cognitive activity remains researcher-centered. This AI use behavior only undertakes simple mechanical tasks, making it difficult to trigger a reconfiguration of the cognitive system (Westover, 2025; Khan & Suhluli, 2025). Its relationship with students’ research ability is weak. In contrast, deeper AI usage behaviors will collaborate with researchers by stimulating higher-order tasks such as reflection (Z. T. Zhang & Reicherts, 2025). This facilitates human–computer collaboration to reconfigure cognitive distribution patterns, thereby improving research capabilities. Based on this, this study proposes the following hypotheses:

**H1.**  *“Technical support AI use” positively impacts the research competence of graduate students.*

**H2.**  *“Text development AI use” positively impacts the research competence of graduate students.*

**H3.**  *“Transformation AI use” positively impacts the research competence of graduate students.*

### 2.2. Mediating Role of Critical Thinking

Critical thinking refers to a learner’s purposeful, self-regulated judgment process that encompasses critical analytical skills, open-mindedness, and a disposition to use critical thinking (Facione, 1990). In the context of the increasing integration of generative AI into scientific research, the role of critical thinking is becoming more and more important. On the one hand, AI-generated content is closely related to the critical thinking skills of college students in open learning environments (W. Zhang & Liu, 2025). The process of revising AI-generated text helps to activate learners’ metacognitive skills and reflective frameworks, which in turn may facilitate the active monitoring of their own thought processes (S. Wang et al., 2025). On the other hand, over-reliance on AI is linked to the degradation of critical thinking. Some studies have shown an inverted U-shaped relationship between the frequency of AI use and critical thinking, with moderate use of AI optimizing research efficiency and over-reliance inhibiting deep thinking (Octaberlina et al., 2024; Goh et al., 2025). Therefore, it is crucial to guide students to apply and develop critical thinking wisely when using GenAI.

The Generative AI Dialogue Collaboration Model for Critical Dialogue and Competency Training proposed by Rahimi (2025) provides a systematic framework for this. The model emphasizes that students should expand and deepen their critical thinking and communication competencies by engaging in structured conversations with GenAI based on their mastery of these competencies. Therefore, Rahimi (2025) has developed a comprehensive Professional Proposal Competency Framework, a scoring scale, and key guiding questions to help students actively question, validate, revise, and deepen their AI outputs rather than passively accepting the information, thereby training them to become “transformational users” of GenAI. Similarly, M. Luo et al. (2025) found that a human–computer collaboration model in which the AI is responsible for linguistic corrections and the teacher focuses on logical guidance in writing feedback is more effective in facilitating the simultaneous improvement of students’ critical thinking and writing quality. In this system, critical thinking is not only manifested as an intrinsic individual cognitive skill but also throughout the whole process of human–computer dialog, collaboration and reflection interaction.

In addition, critical thinking, as a key cognitive skill for improving research competence (Ibragimova et al., 2024), directly enhances the quality of research decision-making, complex problem solving, and research design rationality through skills of analysis, evaluation, and reasoning. Research has shown that there is a significant positive correlation between critical thinking dispositions and undergraduate research ability (Xiao et al., 2013) and that students with higher critical thinking skills are better able to select and analyze information correctly, which supports their research ability (Zhan et al., 2023). Students using GenAI through critical dialogic strategies are able to acquire, evaluate and integrate information more effectively, which in turn contributes to their research skills in research design, problem solving and academic argumentation (Loon, 2020).

Therefore, we believe that critical thinking mediates the relationship between different GenAI use behaviors and research competence. Based on this, this study proposes the following hypotheses:

**H4.**  *Critical thinking mediates the relationship between “technical support AI use” and graduate students’ research competence.*

**H5.**  *Critical thinking mediates the relationship between “text development AI use” and graduate students’ research competence.*

**H6.**  *Critical thinking mediates the relationship between “transformation AI use” and graduate students’ research competence.*

### 2.3. Moderating Role of Research Self-Efficacy

Research self-efficacy refers to an individual’s confidence in his or her ability to successfully accomplish various tasks related to research (Forester et al., 2004) and is a specific manifestation of self-efficacy in the field of research. The moderating effect of research self-efficacy is manifested at multiple levels. A high level of AI self-efficacy can significantly reduce individuals’ anxiety during AI use, promote acceptance and trust in AI technology, and thus indirectly support individuals’ efficiency and decision-making ability (Zhong, 2023). Among doctoral students and university faculty, research self-efficacy significantly predicts research productivity and can further support their research potential by optimizing the research training environment (Woo et al., 2024). Positive effort beliefs and the intensity of AI use behaviors strengthen research self-efficacy, which in turn directly enhances an individual’s actual research output (K.-R. Jung et al., 2017). Meanwhile, research self-efficacy plays a key role in the relationship between research knowledge and research productivity, and researchers with high confidence in their research competence are better able to translate their knowledge base into actual research results (Amanonce, 2025). Research self-efficacy is an important variable that affects research productivity and research interest, and in the face of AI learning and application situations, research self-efficacy tends to reduce cognitive load and support skill learning and adaptation by increasing confidence (Shahzad et al., 2024).

Therefore, this paper proposes the following hypotheses:

**H7.**  *Research self-efficacy moderates the relationship between “technical support AI use” and research competence.*

**H8.**  *Research self-efficacy moderates the relationship between “text development AI use” and research competence.*

**H9.**  *Research self-efficacy moderates the relationship between “transformation AI use” and research competence.*

Many studies have revealed the potential influence of scientific research self-efficacy on fostering critical thinking in the context of AI application. (Guo et al., 2025). Students with a high sense of research self-efficacy are more inclined to critically evaluate and reflect on the information or suggestions generated by AI, rather than blindly accepting them (Jai Lamimi et al., 2025). They believe that they have the ability to distinguish the accuracy and completeness of information, thus reducing the negative impact of AI. Higher AI self-efficacy urges individuals to participate more actively in AI-related activities, which may play a more active role in using AI to support critical thinking (Dong et al., 2025). Based on self-efficacy theory, which centers on an individual’s belief in their capability to complete tasks, this belief directly influences their cognitive engagement. When researchers have a high sense of research self-efficacy, they are more inclined to transform externally available technical tools and text resources into controllable abilities, so as to carry out deep processing more actively in cognition. Therefore, this paper puts forward the following hypotheses:

**H10.**  *Research self-efficacy positively moderates the relationship between “technical support AI use” and critical thinking.*

**H11.**  *Research self-efficacy positively moderates the relationship between “text development AI use” and critical thinking.*

**H12.**  *Research self-efficacy positively moderates the relationship between “transformation AI use” and critical thinking.*

**Figure 1:**
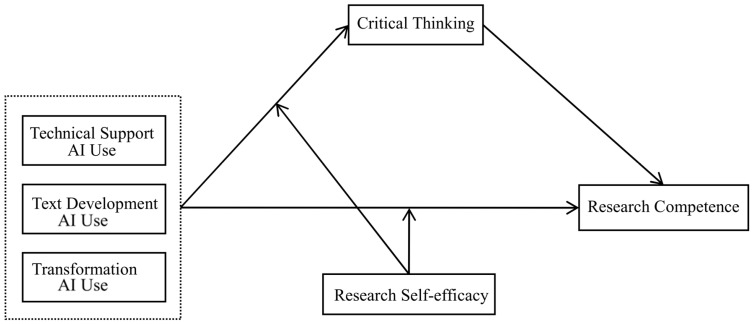
The proposed moderated mediation model.

In summary, this study proposes a moderated mediation model based on distributed cognitive theory and self-efficacy theory. Its main purposes include three aspects: (1) explore the relationship between the use of generative AI and research competence; (2) explore the intermediary role of critical thinking; (3) explore the moderating effect of research self-efficacy. The research framework is shown in Figure 1.

## 3. Materials and Methods

### 3.1. Participants

The study participants were current graduate students at several universities in China, and all of these participants used generative artificial intelligence to assist them in the research process. The study was a minimal-risk anonymous questionnaire survey. It was eligible for ethical review exemption according to the Measures for the Ethical Review of Biomedical Research Involving Humans in China. Specifically, the study was a non-commercial academic study that did not collect any personally identifiable information and did not involve sensitive topics or vulnerable groups. All participation was based on the principle of anonymity and voluntariness in a fully informed manner. The research process strictly adhered to academic ethical norms.

Convenience sampling was used in this study to recruit current graduate students to participate in the survey. Screening questions were set to ensure that respondents had relevant experience in using generative AI techniques. Prior to the start of the study, participants first read the informed consent information and voluntarily checked the box “I have read and understand the above information and I voluntarily agree to participate in this study”. This ensured that all participation was fully informed and voluntary. After participant consent, a screening question section was entered to confirm whether the participant had used a generative AI tool (e.g., ChatGPT, DeepSeek, and Doubao). Only those who confirmed the use of AI tools were eligible to participate in the survey, and those who met the inclusion criteria went on to complete the formal questionnaire.

The questionnaire was conducted through a combination of online and offline methods. The online survey was created electronically through the Questionnaire Star platform and the IP addresses were anonymized. It was distributed on the Little Red Book online platform. The offline survey was conducted in a controlled classroom environment on campus. The researcher distributed QR codes for the paper version of the questionnaire, which were scanned and filled in by the students. To prevent duplicate responses from the same individual, the survey was configured to allow only one submission per registered user. After eliminating the blank questionnaires, questionnaires with repeated responses, and invalid questionnaires with missing values, 522 valid questionnaires were retained, with a response rate of 96.08%, and basic participant information is shown in Table 2.

### 3.2. Measures

In order to ensure the scientific rigor, reliability, and validity of measurement tools, this study constructed and tested the relevant measurement scales around four core variables: research competence, generative AI use, critical thinking, and research self-efficacy. Except for the self-developed scale for generative AI use, all other variables were based on established scales, and the scale development process included key steps such as item design and pre-experiment.

In terms of scale scoring, this study adopted 5-, 6- and 7-point Likert scales for different variables. Specifically, the Research Ability Scale and the Generative AI Use Scale were scored on a 5-point scale. The Critical Thinking Scale was scored on a 7-point scale to capture the continuous changes in individual thinking tendencies in a more detailed way. The Research Self-Efficacy Scale followed the 6-point version that has been widely used in the literature. This choice of differentiation was based on two main considerations. The first was to prioritize the reliability and validity of the scale by directly adopting the mature scale version. Second, the scoring levels were adjusted to optimize the measurement effect according to the measurement refinement needs of different concepts.

This was despite the fact that the different scale scoring ranges may result in means and variances of raw scores that are not directly comparable. However, during the data analysis phase, all variables were processed by calculating question item means and included as continuous variables in subsequent regression analyses. The final reported results were all standardized path coefficients, reflecting the relative change in the relationship between variables rather than absolute value comparisons. Thus, potential problems associated with differences in the number of points on different scales were effectively overcome. The specific measurement instruments were as follows:

#### 3.2.1. Generative AI Use

Generative AI usage behavior included three dimensions: technical support AI use, text development AI use, and transformation AI use. First, this study referred to the scale framework of Jin et al. (2025a) and constructed the initial scale based on a literature review and qualitative analysis. Technical support AI use included three questions, including “I use AI to assist in the collection and processing of research data.” Text development AI use included three items, including “I use AI to polish language expression and make content modifications”, and transformation AI use included three items, including “I use AI to facilitate reflection.” Subsequently, the study was pre-tested to test the reliability of the scales. The results of the item analysis showed that the extreme group *t*-tests for all items were significant (*p* < 0.001), and the correlation between item and total scores showed that the Pearson correlation coefficients ranged between 0.424 and 0.643 (*p* < 0.001), which indicated good item discrimination.

To test the construct validity of the scale, the total sample (*n* = 522) was randomly divided into two parts in this study. First, an exploratory factor analysis (EFA) was conducted in SPSS 27 using the first part of the sample (*n* = 211). The results showed a KMO value of 0.781 and a significant Bartlett’s test of sphericity (χ^2^ = 777.752; df = 36; *p* < 0.001), indicating that the data were suitable for factor analysis (Osborne, 2008). Using principal component analysis with maximum variance rotation, three common factors with eigenvalues greater than 1 were extracted with a cumulative explained variance of 73.86%. The loadings of each question item on its corresponding factor ranged from 0.742 to 0.883, with no significant cross-loadings and a clear factor structure. Subsequently, a confirmatory factor analysis (CFA) was conducted in Amos 27 using the second part of the sample (*n* = 311). The model fit indices are shown in Table 3, with all indices meeting good fit criteria. Further examining the convergent validity of the measurement model, the combined reliability (CR) of the latent variables ranged from 0.842 to 0.863, which were all greater than the criterion of 0.70 (Anderson & Gerbing, 1988); average variance extracted (AVE) ranged from 0.640 to 0.678, values that are all greater than the 0.50 criterion (Hair et al., 2006), which suggests that the scale has good convergent validity. Furthermore, the reliability analysis showed that Cronbach’s alpha coefficients of the three types of AI use behaviors, namely, technical support AI use, text development AI use, and transformation AI use, were 0.837, 0.830, and 0.854, respectively, which all reached a high level of internal consistency. The detailed items are presented in Table A1 of Appendix A.

#### 3.2.2. Research Competence

Research competence in this study refers to a process of inquiry that is systematic, rigorous, methodologically controlled, transparent, and aimed at creating new knowledge (Böttcher & Thiel, 2017). Referring to the R-Comp scale developed by Böttcher, this study used a cognitive perspective to measure graduate students’ research competence, which was categorized into five dimensions: review of the current state of research, methodological skills, reflection on research results, communication skills, and content knowledge. The results of the reliability test showed that the Cronbach’s alpha coefficients of the dimensions were 0.874, 0.904, 0.861, 0.865, and 0.903, which were all higher than 0.8, indicating that the scale had good internal consistency. For detailed items, see Table A2 in Appendix A.

#### 3.2.3. Critical Thinking

The critical thinking in this study adopts the relevant concepts proposed by Byrnes (Byrnes & Dunbar, 2014), including three aspects: critical analytical ability, which refers to the methodical collection and analysis of background information related to the problem and the assessment of the truth of the problem; open-mindedness, which refers to one’s openness to the problem and the collection and analysis of information that is not confined to one’s own inherent point of view; and the tendency to use critical thinking, which refers to the use of critical thinking, i.e., the effort required by the individual to apply critical thinking, and represents the individual’s willingness to apply critical thinking. In order to guarantee the cultural adaptability of the measurement tool, this study used the Chinese Critical Thinking Scale developed by Hou based on the Byrnes’ model for measurement (Hou et al., 2022), and the reliability analysis showed that the Cronbach’s alpha coefficients of the scale’s three dimensions of skill, openness, and application were 0.893, 0.899, and 0.809, respectively, which all reached a high level of internal consistency. Table A3 in Appendix A presents the detailed items.

#### 3.2.4. Research Self-Efficacy

Research self-efficacy in this study refers to the degree of confidence that the subject has in his or her ability to successfully accomplish the research task (Yang et al., 2019). The Research Self-Efficacy Scale developed by Y. Zhang et al. (2013), which includes items such as “I am confident that I can handle the difficulties that arise in the research process”, was used. The reliability test indicated that the scale had a Cronbach’s alpha of 0.914, demonstrating high internal consistency. See Table A4 in Appendix A for the detailed items.

### 3.3. Data Analysis

The data were analyzed using SPSS 26.0 and the macro program PROCESS v3.4.1. Descriptive statistics and correlation analysis were conducted using SPSS 26.0. PROCESS v3.4.1 was used to examine the mediating and moderating models. With technical support AI use, text development AI use, and transformation AI use as independent variables, this study sequentially tested a mediation model mediated by critical thinking and examined the moderating role of research self-efficacy in each model, respectively. Bootstrap sampling was performed with 5000 replications, and the confidence interval was set at 95%. The relationships between research competence and the three types of AI use (for technical support, text development, and transformation) were examined using Pearson correlation coefficients. On this basis, a moderated mediation model was employed to assess the mediating role of critical thinking in the relationship between GenAI use and research competence, as well as the moderating role of research self-efficacy.

## 4. Results

### 4.1. Graduate Students’ Use of Generative AI

In order to gain insight into students’ use of generative AI in research, this study conducted descriptive statistics on the length of use, frequency of use, and length of research experience. As shown in Table 4, 77% of the graduate students had been using it for more than 3 months, with the highest proportions in “more than one year” (26.8%) and “three to six months” (27%), indicating that most students had used AI-assisted research for an extended period of continuity. In terms of the frequency of use, “regularly use” (51%) and “occasionally use” (26.2%) have the highest proportions, indicating that AI had been applied to scientific research behaviors and accounts for a high percentage, and “rarely use” (3.1%) accounts for a low percentage, indicating that AI use in scientific research was widespread in the sample. In terms of the length of research experience, 73.6% of the students had more than one year of research experience, indicating that the sample had substantial research experience.

### 4.2. Descriptive Statistics and Correlation Analysis

Table 5 presents the means, standard deviations, and correlations between the research variables. First, the mean values of the variables showed that transformation AI use (M = 3.97) was the highest and text development AI use was the lowest (M = 3.64); this suggests that graduate students are more inclined to use transformation and upgrading of AI in scientific research, rather than technical application AI or text development AI. In addition, the mean value of research competence is 3.79, the mean value of critical thinking is 4.82, and the mean value of research self-efficacy is 4.45, all of which are relatively high, indicating that graduate students as a whole have high research competence, critical thinking, and research self-efficacy in the context of generative AI intervention in research.

Secondly, in terms of the correlation between variables, technical support AI use, text development AI use, and transformation AI use are all significantly positively correlated with research competence (r = 0.354, 0.394, and 0.391, respectively); critical thinking has a strong positive correlation with research competence (r = 0.413), and research self-efficacy is highly positively correlated with research competence (r = 0.350). Technical support AI use, text development AI use, and transformation AI use also have significant positive correlation with critical thinking (r = 0.289, 0.281, 0.296, respectively). The correlations between the variables reached statistical significance (*p* < 0.05), which provides a prerequisite for further analyses.

### 4.3. Testing the Main Effects of Generative AI Usage Behaviors on Research Competence

This study used a multiple linear regression analysis to explore the association between generative AI usage behaviors (technical support AI use, text development AI use, and transformation AI use) and research competence. Data were analyzed using SPSS 24.0 statistical software to construct multiple regression models. A multicollinearity diagnosis was conducted to assess whether multicollinearity affected the stability of the regression results. The analysis results showed that the variance inflation factor (VIF) of each variable ranged from 1.191 to 1.227, values that are all lower than the critical value of 5.0 proposed by Hair et al. (2014), indicating that the regression models constructed in this study did not have serious multicollinearity problems and that the models were appropriately specified.

As shown in Table 6, gender and age were not significantly associated with research competence. Among the variables, the use of AI-assisted research for more than 6 months significantly and positively predicted research capacity. H1, H2, and H3 examined the direct effects of AI use in the technical support category, AI use in the text development category, and AI use in the transformation and upgrading category by postgraduate students on research competence. The results of the analysis showed that technical support AI use (β = 0.183; *p* < 0.001), text development AI use (β = 0.265; *p* < 0.001) and transformation AI use (β = 0.251; *p* < 0.001) are all positively associated with research competence and that H1, H2, and H3 are valid.

### 4.4. Mediation Effect Test

In this study, the data were processed using Model 4 of the Process plug-in, and three mediation models were constructed to test the mediating role of critical thinking by incorporating technical support AI use, text development AI use, and transformation AI use into the model through the bootstrap method, respectively.

As shown in Table 7 and Table 8, technical support AI use, text development AI use, and transformation AI use all had a significant positive effect on research competence. The direct effect of technical support AI use on research competence was significant (β = 0.076; *p* < 0.01), and after adding the mediating variable of critical thinking, technical support AI use still had a significant positive effect on research competence (β = 0.099; *p* < 0.01). Furthermore, the positive effect of technology application on critical thinking was significant (β = 0.112; *p* < 0.01). In terms of the mediating path, the indirect effect of critical thinking was 0.023, with a 95% confidence interval of [0.017, 0.070], which did not include zero. Thus, H4 was supported.

Text development AI use had a significant positive effect on research competence (β = 0.130; *p* < 0.001), and with the addition of the mediating variable of critical thinking, text development AI use still had a significant positive effect on research competence (β = 0.156; *p* < 0.001). Text development AI use also had a significant positive effect on critical thinking (β = 0.123; *p* < 0.001), and in terms of the mediating path, the indirect effect of critical thinking was 0.026, with a 95% confidence interval of [0.019, 0.073], Therefore, H5 was supported.

Transformation AI use had a significant positive effect on research competence (β = 0.140; *p* < 0.01), and the relationship remained positive and significant after the inclusion of the critical thinking (β = 0.171; *p* < 0.001); transformation AI use also had a significant positive effect on critical thinking (β = 0.153; *p* < 0.001), and the indirect effect of its confidence interval similarly did not contain zero (0.020, 0.078). Therefore, H6 was supported. In summary, all three types of AI use had a significant positive indirect effect on research ability by enhancing critical thinking, and hypotheses H4, H5, and H6 are supported. It is important to note that despite the statistical significance of these mediating pathways, their indirect effect values are relatively small. In this study, the mediating role played by critical thinking was stable but limited in extent. 

### 4.5. Moderated Mediation Effect Test

Based on the mediation model, three regression models were constructed by adding research self-efficacy as a moderating variable. The moderated mediation analysis was conducted using Model 8 of Hayes’ SPSS macro, with the results presented in Table 9 (Hayes, 2009). Research self-efficacy exhibited a significant moderating effect on all three path coefficients.

Specifically, in the model where technical support AI use affects research competence, the interaction term between research self-efficacy and technical support AI use was a significant positive predictor of critical thinking (β = 0.062; t = 2.969; *p* < 0.01), as well as a significant positive predictor of research competence (β = 0.103; t = 6.688; *p* < 0.001). This suggests that research self-efficacy moderates not only the mediating path of “technical support AI use → critical thinking” but also the direct path of “technical support AI use → research competence.” H7 and H10 were supported. Similarly, in the text development AI use model, the moderating effects of the interaction term on critical thinking (β = 0.076; t = 3.197; *p* < 0.01) and research competence (β = 0.094; t = 5.339; *p* < 0.001) were also significant. H8 and H11 were supported. In the transformation AI use model, the interaction term’s moderating effects on critical thinking (β = 0.066; t = 2.685; *p* < 0.01) and research competence (β = 0.122; t = 6.808; *p* < 0.001) were statistically significant, supporting H9 and H12.

**Figure 2:**
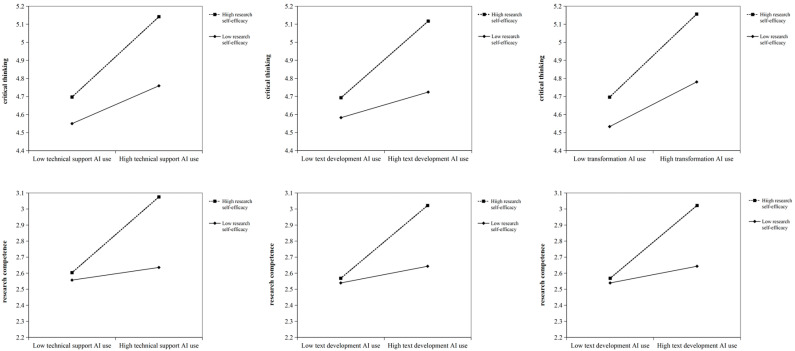
The moderating path coefficient.

To further validate the moderating effect of research self-efficacy, we conducted a simple slope analysis, categorizing research self-efficacy into low (M − 1SD), medium (M), and high (M + 1SD) levels. As presented in Table 10, the results indicated a consistent positive enhancement pattern of the moderating effect across all three paths. Specifically, in the path from technical support AI use to research competence, when research self-efficacy was low, the direct effect of technical support AI use was low (b = 0.041; *p* > 0.05) and the indirect effect was not significant (b = 0.024; *p* > 0.05); in the path from text development AI use to research competence, when research self-efficacy was low, both the direct and indirect effects were small, with the indirect effect not reaching significance (b = 0.017; *p* > 0.05). As research self-efficacy increased to medium and high levels, both the direct effects (increasing to 0.179 and 0.248, respectively) and indirect effects (increasing to 0.041 and 0.050, respectively) were significantly enhanced. In the paths involving text development AI use and transformation AI use, as research self-efficacy increased, both the direct effect of the independent variable on research competence and the indirect effect through critical thinking increased significantly. Figure 2 shows the moderating effect of research self-efficacy across the aforementioned paths.

In summary, research self-efficacy demonstrated a consistently positive moderating effect across all pathways. Specifically, it positively moderated the strength of the effect of the independent variable on the mediator variable (critical thinking) and also positively moderated the strength of the direct effect of the independent variable on the outcome variable (research competence).

## 5. Discussion

### 5.1. Research Findings

In the digital era, the application of generative AI tools in scientific research is becoming more and more widespread, and its impact on graduate students’ scientific research ability as a key tool to assist scientific research has triggered a wide range of concerns in the academic community. Based on cognitive distribution theory and self-efficacy theory, this study explores the relationship between generative AI use behaviors (technical support, text development, and transformation) and graduate students’ research competence through empirical research methods and analyzes in depth the mediating role of critical thinking and the moderating role of scientific research self-efficacy in this relationship. The findings of the study are summarized in the following three areas.

First, the results of the study show that all three AI use behaviors were positively associated with research competence, with the strongest correlation for text development type and the weakest for technical support type.

Distributed cognition theory suggests that cognition is not enclosed in an individual’s brain but is distributed in a functional system composed of individuals, others, and external representations such as various tools and symbols (Zhou & Fu, 2002). Basic technical AIs (e.g., translation and programming) are mainly used as efficiency tools to share mechanical tasks (X. Zhang & Chen, 2025); text development AI assists researchers in processing information and releasing cognitive resources for integrating scientific research tasks, whereas transformation AI use (e.g., inspired reflection and paradigm exploration) focuses on the thought reconstruction and innovation dimensions. Students engage in purposeful, reflective conversations with GenAI to further expand and deepen competencies (Rahimi, 2025).

Although technical support AI is associated with scientific research ability, long-term blind acceptance may contribute to technology dependence (Goh et al., 2025). Researchers should prioritize transformational AI use over relying on basic technology support, thereby transforming AI into a cognitive partner that inspires research innovation. Educators can consciously guide students to move beyond instrumental use to higher-order collaboration centered on reflection.

Second, critical thinking plays a positive mediating role between all three AI use behaviors and scientific research capability, which reflects the importance of critical thinking as a core competency in the integration of AI into scientific research. This suggests that when AI tasks involve complex thinking, students rely on trainable critical skills to make judgments and integrate AI output (Zhao et al., 2025).

The total effect of the three types of AI use behaviors on research competence is higher than the direct effect. This suggests that critical thinking may have increased the positive association between AI use behavior and scientific competence (Qu et al., 2025). This result is highly consistent with Rahimi’s (2025) synergistic framework, which was designed to provide a competency framework with accompanying scoring scales that provide specific strategies for students to practice critical thinking in conversations with AI through follow-up questions of different dimensions.

However, the amount of mediating effect of critical thinking in all three types of AI use was small. This reflects the fact that in real research education scenarios, the enhancement of student competence is the result of multiple factors (mentorship, academic training, personal motivation, etc.); AI tools and critical thinking are only part of the picture. Therefore, educators should not exaggerate the role of AI as a technological tool but rather view it as a helpful and integrable adjunct. In future pedagogical interventions, synergistic approaches could be systematically applied to develop students’ ability to engage in critical dialogs using specific frameworks and tools and discern the information and ideas provided by AI, to cultivate their own competence and learning.

Third, research self-efficacy moderated both the mediating path of “AI use → critical thinking” and the direct path of “AI use → research ability” in the process of the three types of AI use behaviors affecting research competence. Specifically, the higher the research self-efficacy, the stronger the positive effects of these two pathways were. Research self-efficacy potentially moderates the relationship between AI use and critical thinking, with the positive association strengthening at higher levels of self-efficacy (Y. Li et al., 2024). Meanwhile, the direct relationship between AI use and research competence was also stronger (Shahzad et al., 2024). This dual moderating effect was most pronounced in the transformational and upgrading category of AI use pathways. Higher scientific self-efficacy showed a stronger moderating effect on the positive relationship between AI use in the transformation and upgrading category and critical thinking and scientific research ability (Ofem et al., 2024). When AI tasks involve higher-order thinking transitions, students with high research self-efficacy are more likely to initiate critical thinking and exploration of AI-generated content.

### 5.2. Practical Implications

#### 5.2.1. From Tool Use to Cognitive Transformation

The use of AI for transformational upgrades and text development categories as a collaborative partner, not just an aid, should be promoted. The results of the study suggest that graduate students’ AI use behavior beyond tool attributes shows a stronger positive association with their research capabilities. However, frequent or uncritical use of generative AI may foster patterns of dependency (Goh et al., 2025). Therefore, while promoting transformation AI use, universities need to be wary of the long-term risks of over-reliance and prevent AI use from undermining student autonomy.

First of all, colleges and universities and tutors should regard generative AI tools not only as technical aids but also as an important carrier to promote the transformation of scientific research thinking, and institutions and teachers should guide students to gradually realize the transition from “tool use” to “cognitive enhancement” (F. Li et al., 2025). In seminars, students could be encouraged to use AI to conduct ethical reasoning from multiple perspectives or to question the existing theories critically, so as to exercise their in-depth thinking and cognitive development. At the same time, critical assessment of AI output could be emphasized to avoid relying on AI as a substitute for independent thinking, thus balancing the relationship between short-term efficiency and long-term capacity development.

Secondly, colleges and universities could systematically integrate the application scenarios of transformation AI use into their courses and scientific research training and offer thematic workshops such as “AI Collaborative Research Workshop Based on the Competency Framework”, “AI and Scientific Research Ethics” and “Generative AI-driven Critical Thinking Training” (Rahimi, 2025). This will help postgraduates learn how to utilize AI to inspire innovation and optimize research frameworks. Finally, universities may consider setting up interdisciplinary AI innovation platforms to support graduate students in applying such structured and collaborative approaches in real research projects and exploring the deep integration of AI across different disciplines (Anwar, 2025), to drive the shift from technical support AI use to transformation AI use.

#### 5.2.2. Leveraging Critical Thinking

Critical thinking, deepening the efficacy of using generative AI, should be cultivated. Research findings indicate that the beneficial effects of the three types of AI use are closely linked to the systematic cultivation of students’ critical thinking. Therefore, universities and tutors could emphasize the linking role of critical thinking in the AI-enabled research process and actively introduce an actionable teaching framework to systematically cultivate this ability. For example, critical thinking training could be integrated into teaching through collaborative approaches (Rahimi, 2025) to support its use as a cognitive motivator for the development of research skills.

First of all, critical thinking serves as an important correlation between the use of AI for technical support and the development of research skills. Colleges and universities could try to open “AI and Critical Thinking” thematic courses, guide students to examine the logic and reliability of AI-generated content through case study teaching, and set up questioning and verification links to help graduate students establish the habit of scrutinizing the technical operation. This helps to reduce students’ direct dependence on AI results and facilitate the shift from “tool use” to “mindset enhancement” (Rolf, 2025). In addition, this type of training should explicitly discuss dependency risks, such as through Goh et al.’s (2025) scale tool, to help students self-assess their usage patterns and develop healthy usage habits.

Secondly, in the use of AI for text development, critical thinking plays a mediating function between in-depth processing and content reconstruction. Instructors can design problem-oriented writing tasks, guiding graduate students to think critically and innovatively integrate content based on the use of AI to generate literature reviews or experimental protocols. Such training helps students to critically select and optimize AI content (Singh et al., 2025) and cultivate independent thinking and academic creativity. Finally, critical thinking plays a mediation role in transformation AI use. Universities can organize interdisciplinary AI seminar workshops to encourage graduate students to use AI tools for paradigm exploration in complex scientific research scenarios (Drosos et al., 2025). However, it is important to note that over-reliance on AI may weaken self-conceptual clarity, and therefore use should be balanced with autonomous reflection.

It is also worth noting that the pathways of generative AI’s influence on research capacity may be diversified. Research suggests that the effects of AI tools may also be realized through emotional factors such as subjective well-being (H. Li et al., 2023). For example, the immediate interaction provided by AI helps to alleviate loneliness and stress during the research process and provides the necessary psychological energy for scientific research (Hu et al., 2025). This creates favorable conditions for accomplishing research tasks. Therefore, when discussing the impact of AI-enabled research, in addition to focusing on cognitive ability factors such as critical thinking, underlying emotional factors such as happiness need to be taken into account.

#### 5.2.3. Building Research Self-Efficacy

The results of the study indicate that research self-efficacy has a positive moderating effect in the relationship between the three types of AI utilization styles and critical thinking and research competence. College supervisors could take the enhancement of research self-efficacy as a potential supporting factor for AI-enabled research and could contribute to the confidence and ability of graduate students to use AI technology through systematic interventions.

First, for students who tend to prefer technical support AI use, colleges and universities could try to establish a tiered training mechanism to provide more detailed generative AI operation guidance and demonstrations of successful cases for students with a low sense of efficacy and help students accumulate successful experiences in the gradual completion of tasks by setting up task modules from easy to hard. This will help them gradually build confidence in accomplishing their research tasks. Second, tutors could incorporate the use of AI in their research practice to develop students’ confidence, for example by guiding students to take the lead in selecting and evaluating AI tools in their research projects and encouraging them to evaluate the results with professional judgment. This will help to increase their research confidence and sense of research agency. Finally, universities could consider establishing AI innovation programs. This could encourage high-performance graduate students to take the lead in interdisciplinary AI integration research, strengthen their successful experience and increase their self-confidence through the presentation of milestones and reflection during projects (Ofem et al., 2024).

### 5.3. Limitations and Future Directions

Although this study initially examined the relationship between different generative AI use behaviors and research competence, it still has several limitations that future research could address in the following aspects: First, the self-report questionnaire may affect the accuracy and objectivity of the study. Future research could combine mentor evaluations and quantification of research results or use experimental designs to obtain more objective data to improve the accuracy of the research results. Second, the dynamics of variable relationships need to be tracked over time. On the one hand, this study used cross-sectional data for analysis, which allowed for the identification of associations between variables but made it difficult to establish causality. Thus, there exists the same alternative possibility that students with higher critical thinking and research skills themselves are more inclined toward and adept at extensive and transformative AI use. The causal mechanism underlying this correlation remains to be further verified. On the other hand, third variables such as students’ learning ability, research training environment, mentor’s mentoring style, and resource support may simultaneously affect students’ AI use behavior, critical thinking, and scientific research ability, resulting in a potentially spurious observed association. Future designs such as longitudinal tracking, controlling for key covariates, or experimental interventions are needed to clarify causal mechanisms. Third, there is still room for further expansion of the mediating mechanism. This study mainly focused on cognitive pathways such as critical thinking and information processing style, but emotional and social factors, such as individual emotional state and academic social interactions, may also play an important role in the relationship between AI use and research competence. In the future, variables such as academic pressure and AI anxiety could be introduced to construct a more comprehensive multiple mediation model. Fourth, there are some limitations in sample representativeness. In this study, postgraduate students in Chinese universities were the main survey objects, and there may be differences in the acceptance of generative AI among groups with different cultural backgrounds. Subsequently, the scope of the sample could be expanded to include researchers at different career stages and in different countries to conduct cross-group and cross-cultural comparative studies to improve the generalizability of the findings.

## Figures and Tables

**Table 1:** Academic use of artificial intelligence.

Artificial Intelligence (AI) Use	Specific Usage Behavior	Example (Use GenAI to …)	Representative Study	Corresponding Item
Technical support AI use	Data handling	Analyze data results	(Allen & Mizumoto, 2024)	I use AI to assist in collecting and processing research data.
	Chart creation	Create a three-line table or design layouts for charts and graphs	(Palaniappan et al., 2025)	I use AI to assist in generating visualizations.
	Code writing	Write code or check code accuracy	(Y. Liu et al., 2024)	I use AI to assist in writing or debugging research code
Text development AI use	Framework construction	Create an outline and optimize the research framework	(Gahar et al., 2025)	I use AI to generate a preliminary textual framework or thesis outline.
	Polishing text content	Optimize and enhance textual expression, adjusting according to required language styles	(De Wilde, 2024)	I use AI to polish language and expression and to make content revisions.
	Optimizing writing logic	Adjust the logical structure and optimize the content of the paragraphs to make them fit together	(H. Jung & Park, 2023)	I use AI to optimize sentence structure and improve paragraph logic.
Transformation AI use	Interdisciplinary integration	Expand thinking by integrating content from various fields	(K. D. Wang et al., 2024)	I use AI to assist in promoting interdisciplinary and cross-domain knowledge integration.
	Facilitating deeper thinking	Discuss complex issues and provide solutions and ideas	(Lingard, 2023; Chia & Frattarola, 2025)	I use AI to expand my thinking and construct multidimensional perspectives for analyzing and solving problems.
	Facilitating reflection	Reflect on research limitations or shortcomings	(Chan & Lee, 2025)	I use AI to facilitate reflection.

**Table 2:** Basic information of the sample (*n* = 522).

Variable Name	Category	Number of Respondents	Percentage/%
Gender	Male	206	39.4
	Female	316	60.4
Discipline	Humanities and Social Sciences	250	47.8
	Science and Engineering	210	40.2
	Other	62	11.9
Academic Stage	Master’s	445	85.1
	Doctoral	77	14.7
Research Duration	Less than one year	138	26.4
	1–2 years	251	48
	2–3 years	103	19.7
	More than 3 years	30	5.7

**Table 3:** Summary of goodness-of-fit indices for confirmatory factor analysis model.

	χ^2^/df	RMSEA	CFI	TLI	NFI	IFI	RFI
Index	1.296	0.031	0.975	0.992	0.977	0.965	0.965
Benchmark	<3	<0.08	>0.9	>0.9	>0.9	>0.9	>0.9

**Table 4:** Graduate students’ generative AI usage.

Variable	Item	Frequency (*n*)	Proportion (%)
AI Res. Duration	Less than 1 month	33	6.3
	1–3 months	87	16.7
	3–6 months	141	27
	6–12 months	121	23.2
Usage Freq	Rarely (less than once per month)	16	3.1
	Occasionally (1–2 times per week)	137	26.2
	Regularly (3 or more times per week)	266	51
	Frequently (daily)	103	19.7
Res. Duration	Less than one year	138	26.4
	1–2 years	251	48.1
	2–3 years	103	19.7
	More than 3 years	30	5.7

Notes: AI Res. Duration—Duration of AI-Assisted Research; Usage Freq—Usage Frequency; Res. Duration—Research Duration.

**Table 5:** Descriptive statistics and correlation analysis results.

	Mean	SD	1	2	3	4	5	6
1. TSAU	3.689	0.962	1					
2. TDAU	3.641	0.935	0.326 ***	1				
3. TRAU	3.966	0.803	0.365 ***	0.279 **	1			
4. CT	4.82	0.655	0.289 ***	0.281 **	0.296 ***	1		
5. RSE	4.45	1.127	0.268 ***	0.323 **	0.213 ***	0.274 ***	1	
6. RC	3.792	0.545	0.354 ***	0.394 **	0.391 ***	0.413 ***	0.350 ***	1

Notes: TSAU—Technical support AI use; TDAU—Text development AI use; TRAU—Transformation AI use; CT—Critical thinking; RSE—Research self-efficacy; RC—Research competence; ** *p* < 0.01; *** *p* < 0.001.

**Table 6:** Main effects test results.

Variable		*β*	t	*p*
Gender		0.029	−0.580	0.562
Grade		0.082	−1.179	0.239
AI Res. Duration	1–3 months	0.110	0.935	0.350
	3–6 months	0.200	1.681	0.093
	6–12 months	0.249	1.993	0.047 *
	More than 1 year	0.296	2.350	0.019 *
Usage Freq	Occasionally	−0.074	−0.483	0.630
	Regularly	−0.171	−1.105	0.270
	Frequently	−0.269	−1.650	0.100
Res. Duration	1–2 years	−0.045	−0.705	0.481
	2–3 years	−0.135	−1.733	0.084
	More than 3 years	−0.080	−0.668	0.505
AI use	TSAU	0.183	4.397	0.000 ***
	TDAU	0.265	6.555	0.000 ***
	TRAU	0.251	6.124	0.000 ***

Notes: AI Res. Duration—Duration of AI-assisted research (Ref: Less than 1 month); Usage Freq—Usage frequency (Ref: Rarely); Res. Duration—Research duration (Ref: Less than one year); TSAU—Technical support AI use; TDAU—Text development AI use; TRAU—Transformation AI use; * *p* < 0.05; *** *p* < 0.001.

**Table 7:** Summary of regression coefficients for the mediation analyses of AI use types on research competence (*n* = 522).

	Model 1 CT				Model 2 RC			
	*B*	SE	*t*	*β*	*B*	SE	*t*	*β*
Constant	3.356 ***	0.156	21.473	-	1.478 ***	0.16	9.214	-
TSAU	0.112 ***	0.031	3.646	0.164	0.076 ***	0.023	3.272	0.133
TDAU	0.123 **	0.03	4.022	0.175	0.130 ***	0.023	5.63	0.223
TRAU	0.153 ***	0.036	4.235	0.187	0.140 ***	0.027	5.101	0.206
CT	-	-	-	-	0.209 ***	0.033	6.375	0.251
*R* ^2^	0.152				0.319			
Δ*R*^2^	0.147				0.314			
*F*	*F* (3,518) = 30.895, *p* = 0.000				*F* (4,517) = 60.637, *p* = 0.000			

Notes: This table presents a summary of the results from three separate mediation analyses. For each analysis, one type of AI use (technical support, text development, or transformation) was entered as the independent variable, while the other two variables were included as control variables. Model 1 (outcome: critical thinking); Model 2 (outcome: research competence). All coefficients were adjusted for the covariates. TSAU—Technical support AI use; TDAU—Text development AI use; TRAU—Transformation AI use; CT—Critical thinking; RC—Research competence. ** *p* < 0.01; *** *p* < 0.001.

**Table 8:** Summary of mediation effects for three types of AI use (*n* = 522).

Path	Total Effect	Indirect Effect	Direct Effect	95% Boot CI [LLCI, ULCI]
TSAU→CT→RC	0.099 ***	0.023	0.076 ***	[0.017, 0.070]
TDAU→CT→RC	0.156 ***	0.026	0.130 ***	[0.019, 0.073]
TRAU→CT→RC	0.171 ***	0.032	0.140 ***	[0.020, 0.078]

Notes: TSAU—Technical support AI use; TDAU—Text development AI use; TRAU—Transformation AI use; CT—Critical thinking; RC—Research competence; Boot CI = Bias-corrected bootstrap confidence interval based on 5000 resamples; LLCI = Lower limit confidence interval; ULCI = Upper limit confidence interval. *** *p* < 0.001.

**Table 9:** Results of hierarchical regression analysis for moderating effects.

Variable	RC						CT					
	*β*	*SE*	t	*R* ^2^	Δ*R*^2^	*F*	*β*	*SE*	t	*R* ^2^	Δ*R*^2^	*F*
Model 1: Technical Support AI Use												
TSAU	−0.307 ***	0.065	−4.713	0.333	0.327	F(4,517) = 64.591 ***	0.197 ***	0.029	6.885	0.084	0.080	F(1,520) = 47.406 ***
RSE	−0.261 ***	0.055	−4.709									
TSAU×RSE	0.103 ***	0.015	6.688									
CT	0.213 ***	0.032	6.608									
Model 2: Text Development AI Use												
TDAU	−0.257 ***	0.079	−3.257	0.323	0.316	F(4,517) = 61.666 ***	0.197 ***	0.029	6.667	0.079	0.075	F(1,520) = 44.453 ***
RSE	−0.239 ***	0.065	−3.703									
TDAU×RSE	0.094 ***	0.018	5.35									
CT	0.218 ***	0.032	6.739									
Model 3: Transformation AI use												
TRAU	−0.315 ***	0.077	−4.09	0.351	0.344	F(4,517) = 69.836 ***	0.241 ***	0.034	7.057	0.087	0.084	F(1,520) = 49.796 ***
RSE	−0.364 ***	0.072	−5.093									
TRAU×RSE	0.122 ***	0.018	6.79									
CT	0.204 ***	0.032	6.397									

Notes: TSAU—Technical support AI use; TDAU—Text development AI use; TRAU—Transformation AI use; CT—Critical thinking; RC—Research competence; RSE—Research self-efficacy; *** *p* < 0.001.

**Table 10:** Analysis of the moderating role of research self-efficacy.

Path		RSE	Effect Size	SE	Boot CL
TSAU→RC	Direct Effect	Low (M − 1SD)	0.041	0.025	[−0.009, 0.091]
		Medium (M)	0.179 ***	0.023	[0.133, 0.225]
		High (M + 1SD)	0.248 ***	0.029	[0.192, 0.304]
	Indirect Effect	Low (M − 1SD)	0.024	0.009	[0.008, 0.044]
		Medium (M)	0.041 ***	0.011	[0.023, 0.064]
		High (M + 1SD)	0.050 ***	0.013	[0.028, 0.077]
TDAU→RC	Direct Effect	Low (M − 1SD)	0.057 *	0.028	[0.001, 0.112]
		Medium (M)	0.182 ***	0.024	[0.135, 0.227]
		High (M + 1SD)	0.245 ***	0.029	[0.187, 0.302]
	Indirect Effect	Low (M − 1SD)	0.017	0.01	[−0.003, 0.036]
		Medium (M)	0.038 ***	0.01	[0.019, 0.06]
		High (M + 1SD)	0.050 ***	0.013	[0.025, 0.077]
TRAU→RC	Direct Effect	Low (M − 1SD)	0.093 **	0.028	[0.037, 0.149]
		Medium (M)	0.256 ***	0.028	[0.201, 0.311]
		High (M + 1SD)	0.338 ***	0.035	[0.270, 0.406]
	Indirect Effect	Low (M − 1SD)	0.031 **	0.011	[0.014, 0.055]
		Medium (M)	0.049 ***	0.012	[0.027, 0.074]
		High (M + 1SD)	0.058 ***	0.014	[0.032, 0.087]

Notes: TSAU—Technical support AI use; TDAU—Text development AI use; TRAU—Transformation AI use; RC—Research competence; RSE—Research self-efficacy; Boot CI = Bias-corrected bootstrap confidence interval based on 5000 resamples; LLCI = Lower limit confidence interval; ULCI = Upper limit confidence interval. * *p* < 0.05; ** *p* < 0.01; *** *p* < 0.001.

## Data Availability

The original contributions presented in this study are included in the article. Further inquiries can be directed to the corresponding author.

## Appendix A. Measurement Scales Used in the Survey

**Table A1:** GenAI use scale.

Dimension	Item
Technical support AI use	I use AI to assist in collecting and processing research data.
	I use AI to assist in generating visualizations.
	I use AI to assist in writing or debugging research code.
Text development AI use	I use AI to generate a preliminary textual framework or thesis outline.
	I use AI to polish language and expression and to make content revisions.
	I use AI to optimize sentence structure and improve paragraph logic.
Transformation AI use	I use AI to assist in promoting interdisciplinary and cross-domain knowledge integration.
	I use AI to expand my thinking and construct multidimensional perspectives for analyzing and solving problems.
	I use AI to facilitate reflection.

**Table A2:** Research competence scale.

Dimension	Item
Skills in reviewing the state of research	I know how and where to target a search of the state of research regarding a specific topic.
	I am able to systematically review the state of research regarding a specific topic.
	Based on the state of research, I am able to identify gaps/unaddressed questions for further research.
	I can evaluate the methodological quality of researched findings well.
Methodological skills	I find it difficult to formulate specific research questions/hypotheses.
	I am able to decide which data/sources/materials I need to address my research question.
	I am able to plan a research process.
	I find it difficult to operationalize each step of the research process.
	I find it easy to decide which methods I need to use to examine a specific research topic.
	I am good at judging which method is inappropriate to answer a specific research question.
	I can apply different research methods appropriate to my research question.
	I can confidently apply even complex methods to analyze data/sources/materials.
Skills in reflecting on research findings	I am able to adequately interpret my own research findings by relating them to key theories in the subject area.
	I am able to critically reflect on methodological limitations of my own research findings.
	I am able to reflect on the implications of my own research findings on my discipline.
	I am able to discuss my research findings with regard to their potential applications.
	I am able to critically reflect on the social/ethical implications of my research.
	I am able to take a stand on social/ethical issues of research in my discipline.
	I can apply different research methods appropriate to my research question.
Communication skills	I can write up research findings in accordance with the current conventions in my discipline.
	I am able to write a publication in accordance with the standards of my discipline.
	I find it difficult to write a report that meets the standards of academic writing.
	I am able to prepare research findings for a presentation at a research colloquium.
	I am able to present my research at a scientific meeting in accordance with current standards in my discipline.
Content knowledge	I have a good overview of the main (current) research findings in my discipline.
	I am informed about the main (current) theories in my discipline.
	I am informed about the history of theory/paradigm shifts in my discipline.
	I have a sound knowledge of the main research methods in my discipline.
	I would describe my methodological knowledge as sophisticated and comprehensive.
	I am very familiar with different research methods in my subject area.
	I am informed about the most important national and international academic publication outlets in my discipline.
	I am informed about the standards for academic publications that apply in my discipline.
	I am informed about the standards that apply to the presentation of research findings at congresses and meetings in my subject area.

**Table A3:** Critical thinking scale.

Dimension	Item
Skills	I clearly understand my objectives before taking action.
	I prefer to follow a structured approach when solving problems.
	I value the opinions of my parents and elders when making decisions.
	I adapt my decisions to different situations when facing problems.
	I can distinguish which matters are currently important in my life.
	In addition to course-related reading, I also read extensively beyond the curriculum.
	I carefully consider the validity of the saying, “Everything happens for a reason.”
	I am able to communicate effectively with people who hold different viewpoints.
Open-mindedness	I find it difficult to accept when others disagree with my views or ideas.
	I feel embarrassed if I change my mind midway through a process.
	When tackling tasks, I tend to stick to the current method without considering alternatives.
	Disagreements with others often hinder our communication.
	I often feel overwhelmed when dealing with complex problems.
Application	I frequently share my perspectives on things with others.
	When a new product’s manual is complex and hard to understand, I prefer learning through hands-on operation.
	I usually have backup plans when solving problems.
	Peers often seek my advice when facing difficult decisions.

**Table A4:** Research self-efficacy scale.

Item
I am confident in my ability to excel in scientific research work.
I am confident in handling the challenges that arise during the research process.
I am full of confidence when it comes to conducting scientific research.
